# Genome-wide identification and analysis of the *UBA2* gene family in wheat (*Triticum aestivum* L.)

**DOI:** 10.1186/s12864-025-11352-z

**Published:** 2025-02-22

**Authors:** Juan Li, Chunge Cui, Fengying Han, Jin Liu

**Affiliations:** 1 College of Forestry Engineering, Shandong Agriculture and Engineering University, Jinan, 250100 China; 2 Shanxi Medical University, Taiyuan, 030000 China

**Keywords:** Wheat, RNA-binding protein, UBA2 family, Genome-wide, RNA recognition motif, Subcellular localization

## Abstract

**Background:**

RNA-binding proteins (RBPs) participate in multiple aspects of RNA metabolism, which in turn regulates gene expression, thereby involving in organism growth and development. The UBA2 family, one of the subfamilies of RBPs, has been identified in several plant species. However, few researches have been performed to investigate the role of UBA2 in wheat (*Triticum aestivum*).

**Results:**

In this study, we identified eleven *TaUBA2s* and divided them into three groups according to their domain characteristics. Phylogenetic analysis was conducted to forecast functional similarities among *Arabidopsis*, rice, maize and wheat *UBA2* genes. Members within the same subfamily of TaUBA2 are relatively conserved in terms of protein structure, motifs, and gene structure. Chromosomal location and synteny analysis suggested that the segmental duplication events played important roles during *TaUBA2s* evolution. The cis-acting element analysis showed that *TaUBA2s* were involved in hormone response, development, light response, metabolism, and response to environmental stress. Furthermore, TaUBA2C contains two RNA recognition motifs (RRMs), and the first RRM is responsible for the nuclear speckle formation of TaUBA2C, whereas the two RRMs are necessary for its biological function.

**Conclusions:**

Taken together, our study provides a comprehensive analysis of the *TaUBA2* family in wheat and lays the foundation for the future functional investigations of *TaUBA2s* in wheat growth, development and stress responses.

**Supplementary Information:**

The online version contains supplementary material available at 10.1186/s12864-025-11352-z.

## Background

RNA-binding proteins (RBPs) play important roles in the regulation of gene expression through co-transcriptional and post-transcriptional modification, in eukaryotes. The typical characteristic of RBPs is the inclusion of multiple RNA binding domains (RBDs), such as RNA recognition motif (RRM), zinc finger domain, K homology domain (KH), chloroplast RNA splicing and ribosome maturation domain (CRM), DEAD-box domain, RNA helicase domain, and the Pumilio/FBF domain, of which RRM is the most abundant domain [1]. It is reported that approximately 200 RBPs which contained the classical RRM were identified in the *Arabidopsis* genome [2]. Meanwhile, there were 178 RBPs that contained the classical RRM in barley [3]. Researches have shown that RBPs not only participate in multiple developmental processes, but also respond to various environmental stresses. For example, AtFLK containing three KH domains and AtGRP7, an RBP rich in glycine, can both participate in regulating flowering time in *Arabidopsis* [4–6]. Moreover, some RBPs have been confirmed to play important roles in abscisic acid (ABA) responses [7–9]. In addition, *Arabidopsis* UBP1-associated protein 2 (UBA2) family members encoding heterogeneous nuclear ribonucleoprotein (hnRNP)-type nuclear RBPs are involved in wounding response, and overexpression of each of the three UBA2 members can induce cell death response in *Arabidopsis* plants [10, 11]. Studies have reported that some RBPs play key roles in cold stress responses [12–15]. The AtRBP-DR1 encoding three classical RRMs participates in SA-mediated plant immunity through acting on genes of SA signal transduction [16]. The *Arabidopsis* RBP, AtAGO2, is reported to regulate *Arabidopsis* defense responses against pathogen infection [17]. RNA binding proteins RZ-1B and RZ-1 C in *Arabidopsis thaliana* are involved in plant growth and development by modulating pre-mRNA splicing [18]. Studies have confirmed that RBPs can also play roles in chromatin modification [19, 20]. A subset of RBP family members in *Arabidopsis thaliana* has been well characterized, and numerous RBPs are unique to plants, suggesting that they may possess plant-specific functions [21].

In the *Arabidopsis* genome, 1145 RNA binding proteins are identified, among them, only a small portion perform functional characterization [22]. The UBA2 subfamily encoding hnRNP-type RBPs has three members, UBA2a, UBA2b, and UBA2c, each of the three UBA2 members contains two classical RRMs [2, 23]. It is reported that UBA2a was identified through UBP1 interaction screening experiments, therefore, UBA2a was also known as ‘UBP1-associated protein 2’ [24]. The genes *UBA2b* and *UBA2c* are characterized by their significant sequence homology with *UBA2a* [10, 24]. UBA2a and UBA2b interact with each other, however, neither of them interacts with UBA2c [25]. The three proteins of the *Arabidopsis* UBA2 subfamily are all located in the nucleus, with the difference being that UBA2a and UBA2b are dispersed throughout the nucleus, while UBA2c appears as speckles in the nucleus, interestingly, UBA2a and UBA2b can form speckles in the nucleus with ABA treatment [8–10, 25]. Overexpression of *Arabidopsis* UBA2 proteins causes leaf yellowing and cell death phenotype, similar symptoms are also observed from leaves overexpressing *S. tuberosum* UBA2s (StUBA2s), suggesting that UBA2s play important roles in leaf senescence and defense-responses [11, 26, 27]. Previous study also showed that AtUBA2c directly binds to FLOWERING LOCUS M (FLM), a flowering repressor, to inhibit histone H3K27 trimethylation, which in turn promotes FLM transcription to prevent early flowering [28]. Researches on UBA2 subfamily mainly focused on *Arabidopsis thaliana*, however, few studies have been performed on bread wheat. In previous studies, we identified an RNA binding protein in wheat and named it TaUBA2C, studies have confirmed that TaUBA2C can bind to *TaNPR1*, *TaPR1* and *TaRBOHD* pre-mRNA to regulate these genes expression which in turn modulates H_2_O_2_ production and cell death, thereby participating in Chinese wheat mosaic virus (CWMV) infection [29]. The role of UBA2 subfamily proteins in regulating programmed cell death (PCD) in plants further underscores their significance in plant immunity and development.

Programmed cell death (PCD) is an important mechanism in plant immune that can protect hosts by clearing damaged or pathogen infected cells [30, 31]. PCD, a genetically regulated cell death process, plays crucial roles in plant growth and development through participating in various biotic and abiotic stresses, such as salinity stress, extreme temperature, and pathogen infection, suggesting that PCD is very common in plant stress response [30, 32–35]. During the PCD process, a series of morphological and biochemical features are presented, for example, membrane folds, DNA laddering, and nuclear pycnosis [36, 37]. It is reported that PCD can be activated by heat shock in *Heterosigma akashiwo* cells [38]. In addition, studies have confirmed that PCD induced by high temperatures is closely associated with epigenetic changes in seedling leaves of *Zea mays* [39]. Moreover, many studies have demonstrated that PCD plays an important role in the arms race between plants and pathogens [29, 40].

Wheat is widely cultivated worldwide, and its annual production is of great significance to food security. It is reported that wheat provides 20% of the required calories for humans [41]. With the growth of the global population, the wheat production is expected to increase by 38% to meet people’s demand for food [42]. In previous studies, we identified a member of the wheat UBA2 subfamily, TaUBA2C, and conducted preliminary exploration of its function [29]. However, the phylogenetic and structural features of the wheat UBA2 subfamily have not yet been characterized. In this study, we performed a genome-wide analysis of the wheat UBA2 subfamily, and eleven UBA2 subfamily members were identified in wheat. Subsequently, we comprehensively analyzed UBA2 phylogenetic relationships, conserved domains, protein and gene structures, chromosomal locations, evolutionary patterns, and cis-acting elements. Furthermore, we also analyzed the effects of two RRM domains of TaUBA2C on its subcellular localization and biological functions. Taken together, our study provides valuable information for the subsequent investigation of TaUBA2 family members and may contribute to further researches of TaUBA2s in wheat against multiple stresses.

## Results

### Identification and characterization of UBA2 in *Triticum aestivum*

**Fig. 1:**
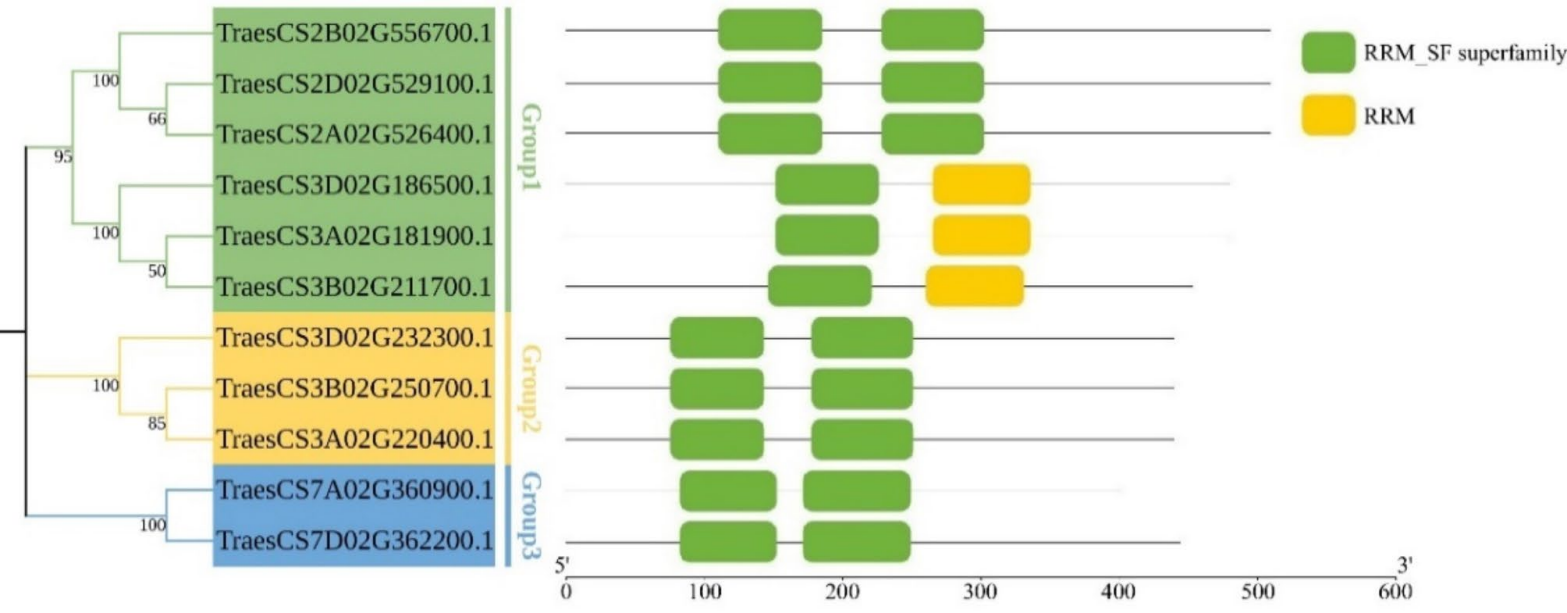
Conserved domain analysis of the *TaUBA2* family. According to the conserved domain analysis, 11 TaUBA2s were divided into three subfamilies: Group1, Group2, and Group3

In order to identify the UBA2 family members in wheat, we performed a whole-genome analysis through the BLSATP approach using UBA2 protein sequences from *Arabidopsis* as queries. All candidates were then submitted to Protein family database (Pfam) for analysis of domain structures. Based on these analyses, a total of 11 UBA2s in wheat were identified. For the convenience of description, the 11 UBA2 gene family members were clustered into 3 groups, namely, Group1, Group2, and Group3, based on their conserved domains and the classification of UBA2s in *Arabidopsis thaliana*. Each group has different conserved domains that support the applicability of such a grouping (Fig. 1). Detailed information about *TaUBA2* gene family, such as gene ID, location, coding sequence (CDS) lengths, amino acid length, molecular weight (MW), isoelectric point (PI), and the number of exons, are provided in Table 1. The CDS of *TaUBA2s* ranged from 1206 (TraesCS7A02G360900.1) to 1530 (TraesCS2A02G526400.1, TraesCS2D02G529100.1, and TraesCS2B02G556700.1) bp in length. Corresponding to the CDSs’ length, *TraesCS7A02G360900.1* encoded the shortest protein (401aa), whereas *TraesCS2A02G526400.1*, *TraesCS2D02G529100.1*, and *TraesCS2B02G556700.1* encoded the longest proteins (509aa). The MW of the TaUBA2s varied from 41.34 (TraesCS7A02G360900.1) to 52.14 (TraesCS2A02G526400.1) kDa. The PI ranged from 4.69 (TraesCS7A02G360900.1) to 8.63 (TraesCS3A02G220400.1, TraesCS3B02G250700.1, and TraesCS3D02G232300.1). The number of exons varied from 1 (TraesCS2B02G556700.1) to 9 (TraesCS7D02G362200.1) (Table 1).

**Table 1:** Detailed information about 11 predicted UBA2 proteins in *Triticum aestivum*

Gene ID	Location	CDS Length (bp)	Size (aa)	MW (kDa)	PI	Exons	Groups
TraesCS7D02G362200.1	7D: 465,332,612 − 465,341,680	1335	444	46.16	4.73	9	Group3
TraesCS7A02G360900.1	7 A: 534,402,505–534,409,817	1206	401	41.34	4.69	6	Group3
TraesCS3B02G211700.1	3B: 250,817,962 − 250,819,389	1362	453	46.83	4.94	2	Group1
TraesCS3D02G186500.1	3D: 172,380,462 − 172,383,336	1443	480	49.43	4.79	2	Group1
TraesCS3A02G181900.1	3 A: 210,258,397 − 210,261,047	1443	480	49.56	4.79	2	Group1
TraesCS3A02G220400.1	3 A: 406,296,887 − 406,300,401	1323	440	43.95	8.63	2	Group2
TraesCS3B02G250700.1	3B: 400,649,155–400,652,733	1323	440	44.01	8.63	2	Group2
TraesCS3D02G232300.1	3D: 319,517,723 − 319,521,926	1323	440	44.03	8.63	3	Group2
TraesCS2A02G526400.1	2 A: 746,753,763 − 746,755,926	1530	509	52.14	6.17	2	Group1
TraesCS2D02G529100.1	2D: 616,569,296–616,574,679	1530	509	52.08	5.99	2	Group1
TraesCS2B02G556700.1	2B: 751,531,003-751,536,058	1530	509	51.94	6.4	1	Group1

### Phylogenetic analysis of the UBA2 proteins

**Fig. 2:**
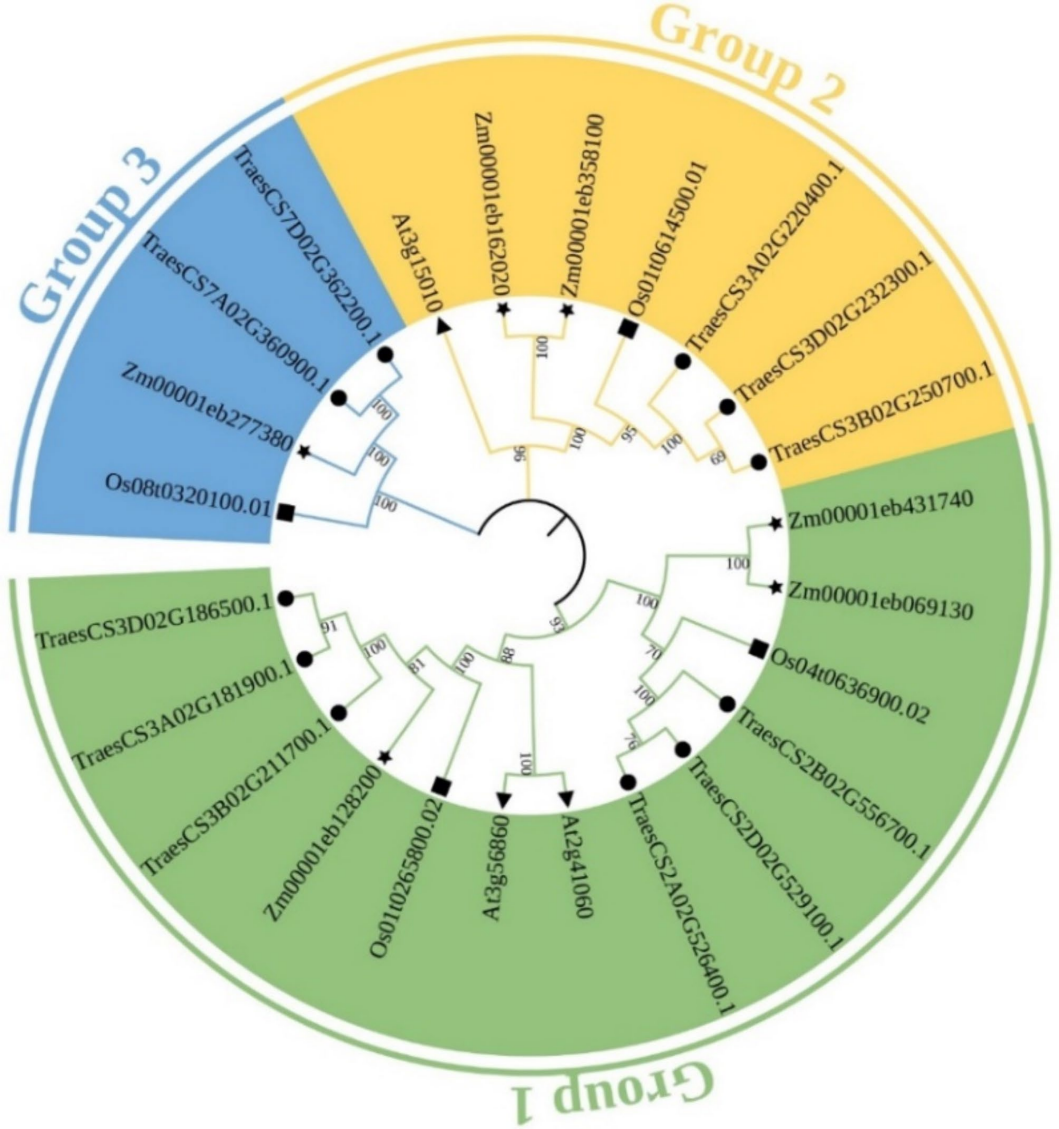
Phylogenetic tree of UBA2 proteins from *Arabidopsis thaliana*, wheat, *Oryza sativa* and *Zea mays* constructed by the neighbor-joining method using MEGA 7.0. The number at node indicates bootstrap value after 1000 iterations. All UBA2 proteins were divided into three subfamilies, and each subfamily is represented by a different color. Triangles, circles, rectangles, and stars correspondingly represent *Arabidopsis*, wheat, rice, and maize

To investigate the evolutionary relationships among UBA2s from different species, 3 *Arabidopsis* (diploid), 11 wheat (hexaploid), 6 maize (diploid), and 4 rice (diploid) UBA2 protein sequences were used to construct a neighbor-joining (NJ) phylogenetic tree using MEGA 7.0 software (Fig. 2 and Table S1). The result showed that UBA2 proteins from the four species were divided into three groups: Group 1, Group 2, and Group 3. As shown in Fig. 2, the phylogenetic analysis showed that TaUBA2s were highly homologous to AtUBA2s, OsUBA2s, and ZmUBA2s. We also found that the phylogenetic distribution of TaUBA2 family members in different branches was not uniform. The Group 1 subfamily contained six members from wheat, the Group 2 subfamily contained three members, whereas the Group 3 subfamily contained only two members. Regardless of species, Group 1 was the largest subfamily, with six TaUBA2s, two AtUBA2s, two OsUBA2s, and three ZmUBA2s. Moreover, each subfamily contained TaUBA2s, OsUBA2s, and ZmUBA2s (Fig. 2).

### Predicted structure analysis of UBA2 proteins

**Fig. 3:**
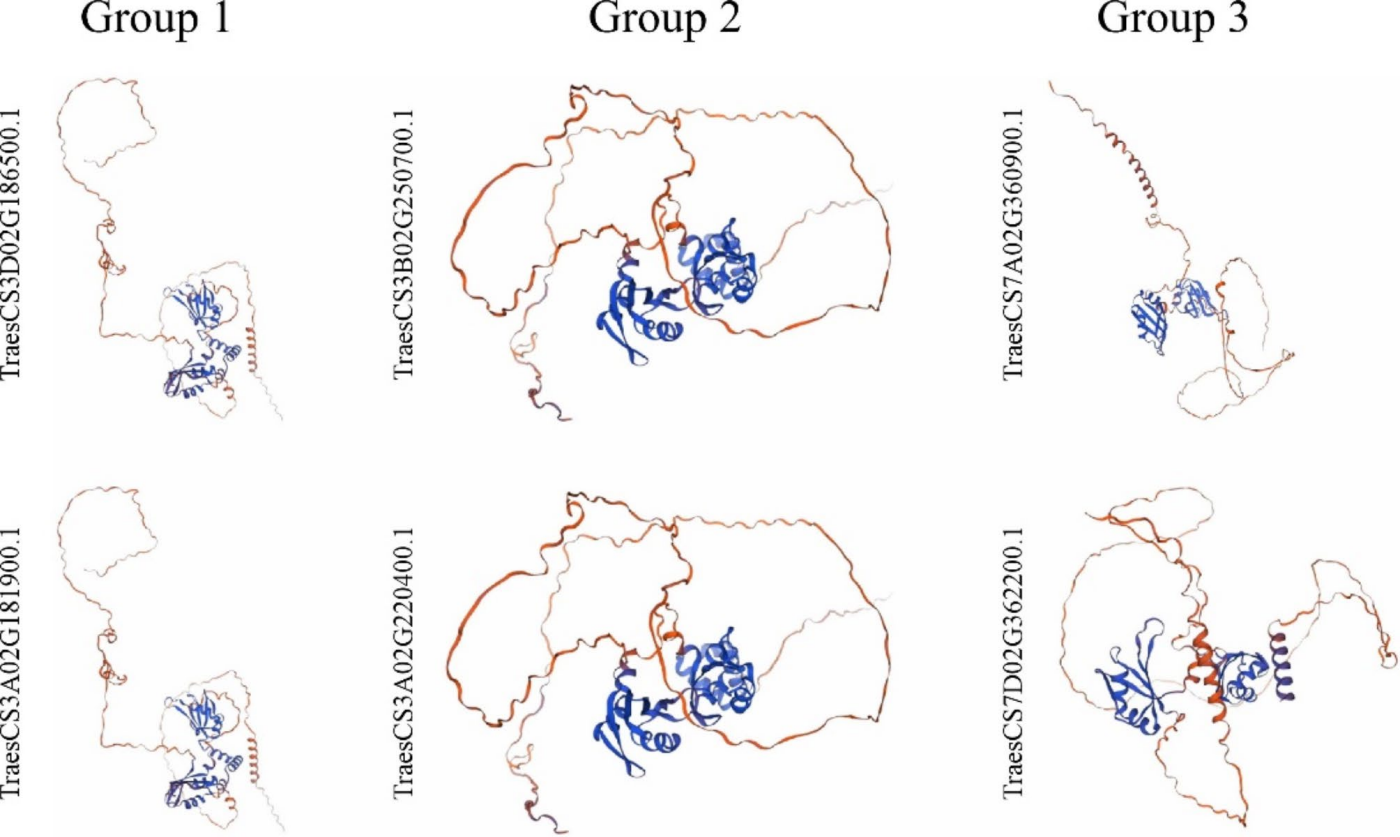
Prediction of the spatial structure of TaUBA2 proteins. The six TaUBA2 proteins were randomly selected from each subfamily. The protein prediction models with high confidence were displayed according to QMEAN and GMQE

It is widely accepted that the spatial conformation and function of proteins are closely related. Homology modelling plays a crucial role in structural biology [43]. To gain insight into the spatial structure of the TaUBA2 proteins, we randomly selected two TaUBA2 proteins from each subfamily to conduct homology modeling by SWISS-MODEL website. Then, we obtained three-dimensional models of the selected proteins (Fig. 3). All selected TaUBA2 proteins could be predicted as models, suggesting that they maintained the integrity of their structure during evolution, which plays a vital role in their function. The result showed that the spatial structure of TaUBA2 proteins belonging to the same subfamily (Group 1 and Group 2) are highly similar, however, the TaUBA2 proteins of Group 3 appeared certain differences in protein structure. Meanwhile, we observed that the protein structures from different groups exhibited significant differences (Fig. 3). These results indicated the structural diversity of the UBA2 family in wheat.

### Gene structures and conserved motifs analyses of wheat UBA2s

**Fig. 4:**
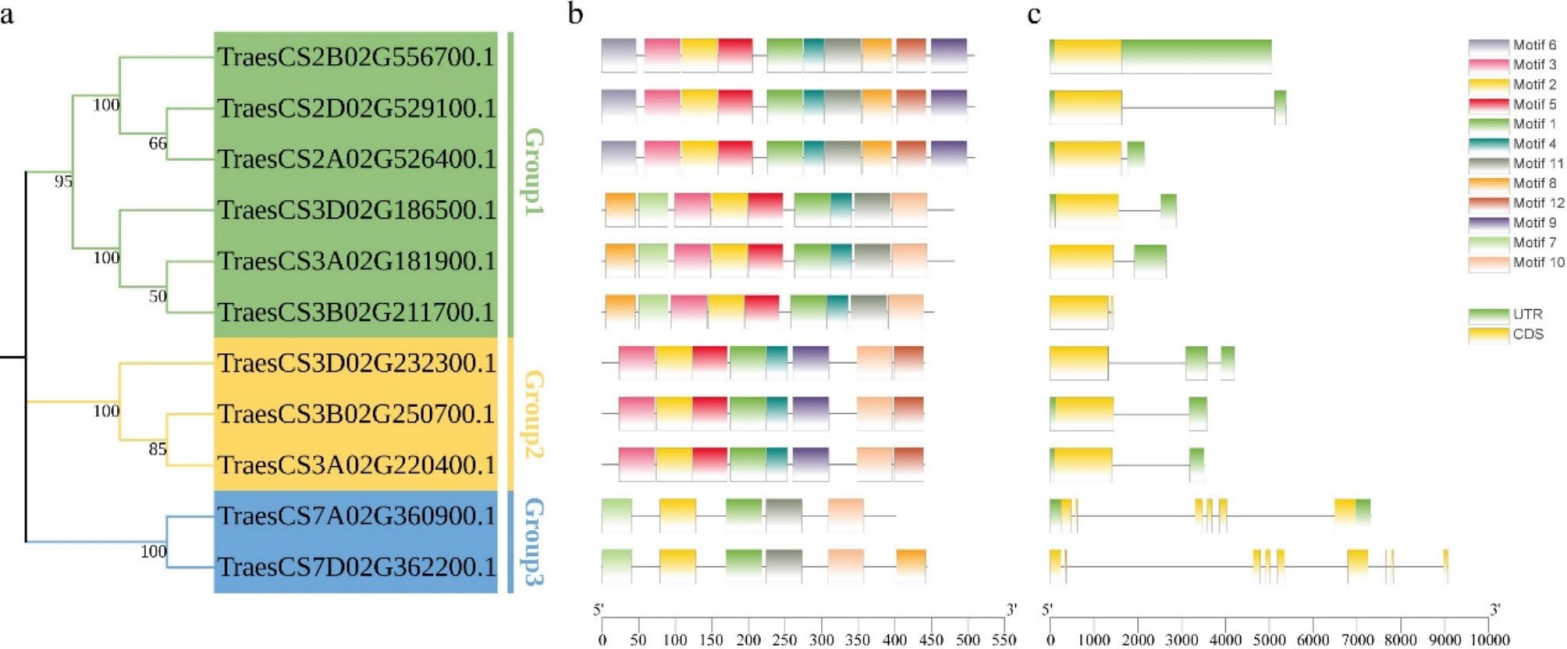
Gene structures and conserved motifs of *TaUBA2s*. (**a**) Phylogenetic tree of TaUBA2s. The phylogenetic tree was constructed using neighbor-joining method with the subfamily names listed on the right. (**b**) MEME motif distribution of TaUBA2 proteins. Different colored frames indicate different motifs. (**c**) Exon–intron structures of 11 *TaUBA2* genes. The yellow frame, grey line, and green frame correspondingly represent exon, intron, and untranslated region

To further investigate the structural features and potential functions of UBA2s in wheat, we uploaded the predicted protein sequences of the 11 TaUBA2 proteins to the MEME website to predict the putative motifs of these proteins. The threshold in the MEME website is set to twelve motifs. The distribution of these putative motifs in TaUBA2 was displayed in Fig. 4b. The motif distribution patterns of TaUBA2 proteins within the same group exhibited a high degree of similarity, suggesting that these proteins may have similar functions [44]. Each TaUBA2 protein of Group 2 contained the same motifs and they were arranged in the same order, indicating that Group 2 subfamily members may have consistency in functionality. The Group 1 subfamily had the largest number of motifs, whereas the Group 3 subfamily had the least number of motifs. Motif 1 and motif 2 were present in all members of TaUBA2 family. Motif 6 was present only in the Group 1 subfamily (Fig. 4b). Since the conserved exon–intron structure of gene family is very important during the evolution of gene families, we analyzed the genomic DNA sequence of *TaUBA2* [45]. As shown in Fig. 4c, the number of exons ranged from 1 to 9. The *TaUBA2s* of Group 1 and Group 2 subfamily had similar exon numbers, whereas, there was a certain difference in the length of their introns. Among all the members of *TaUBA2*, *TraesCS7D02G362200.1* had the largest number of exons. In addition, we also observed that most *TaUBA2s* contained untranslated regions (UTR), except for *TraesCS3B02G211700.1* and *TraesCS7D02G362200.1* (Fig. 4c).

### Chromosomal locations and synteny analysis of *TaUBA2s*

**Fig. 5:**
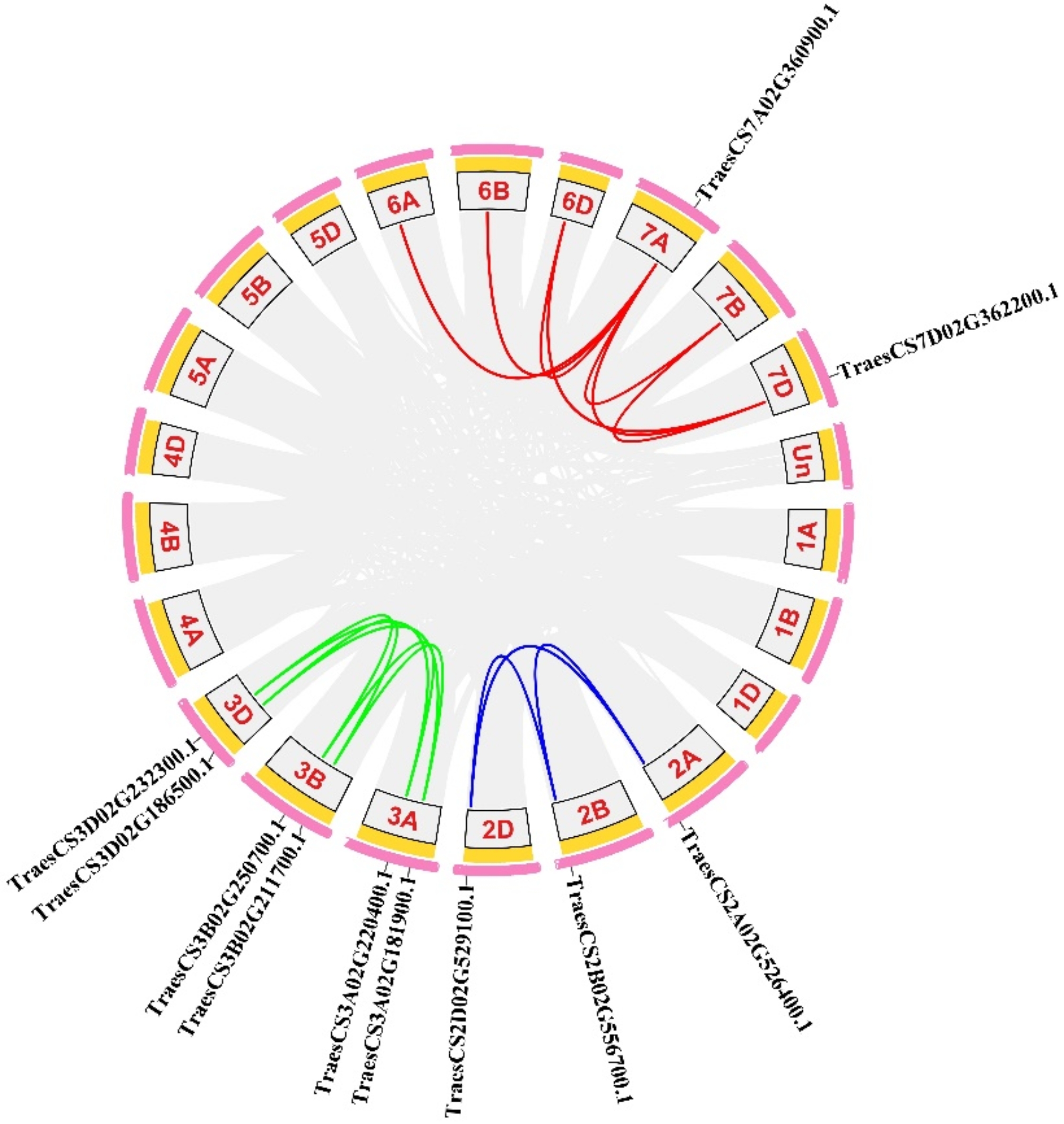
Chromosomal distribution and duplication events analysis of the wheat *TaUBA2* genes. The duplication events are marked with different colored lines, and the positions of *TaUBA2s* are marked directly on the chromosomes. The graphs of chromosomal location and synteny analysis were generated using TBtools

Since the hexaploid wheat has three sub-genomes (A, B, and D sub-genomes), each gene is able to have orthologues on three homologous chromosomes [46]. To better understand the characteristics of the *TaUBA2* family, chromosomal location analysis was performed using TBtools software. The result showed that 11 *TaUBA2* genes were unevenly distributed on the chromosomes of the wheat genome. The A, B, and D sub-genomes contained four, three, and four *TaUBA2* gene family members, respectively. The chromosome 2 contained three *TaUBA2* gene family members, the chromosome 3 had six *TaUBA2* gene family members, and two *TaUBA2* genes were detected on the chromosome 7. Meanwhile, we observed that no *TaUBA2* gene family members were found on chromosome 1, 4, 5, 6, and unknown wheat chromosome (Figure S1). Taken together, there is no significant correlation between the distribution of *TaUBA2s* and the distribution of wheat genes. Tandem and segmental duplications play an important role in the gene family expansion of plants [47]. To investigate the duplication relationship of *TaUBA2* gene family members during evolution, we conducted synteny analysis using TBtools software. The result showed that among the 11 *TaUBA2* gene family members, we identified 10 collinear *TaUBA2* gene pairs, suggesting that segmental duplications were important for the expansion of *TaUBA2* family (Fig. 5).

### Analysis of *TaUBA2* paralogs and orthologs

**Fig. 6:**
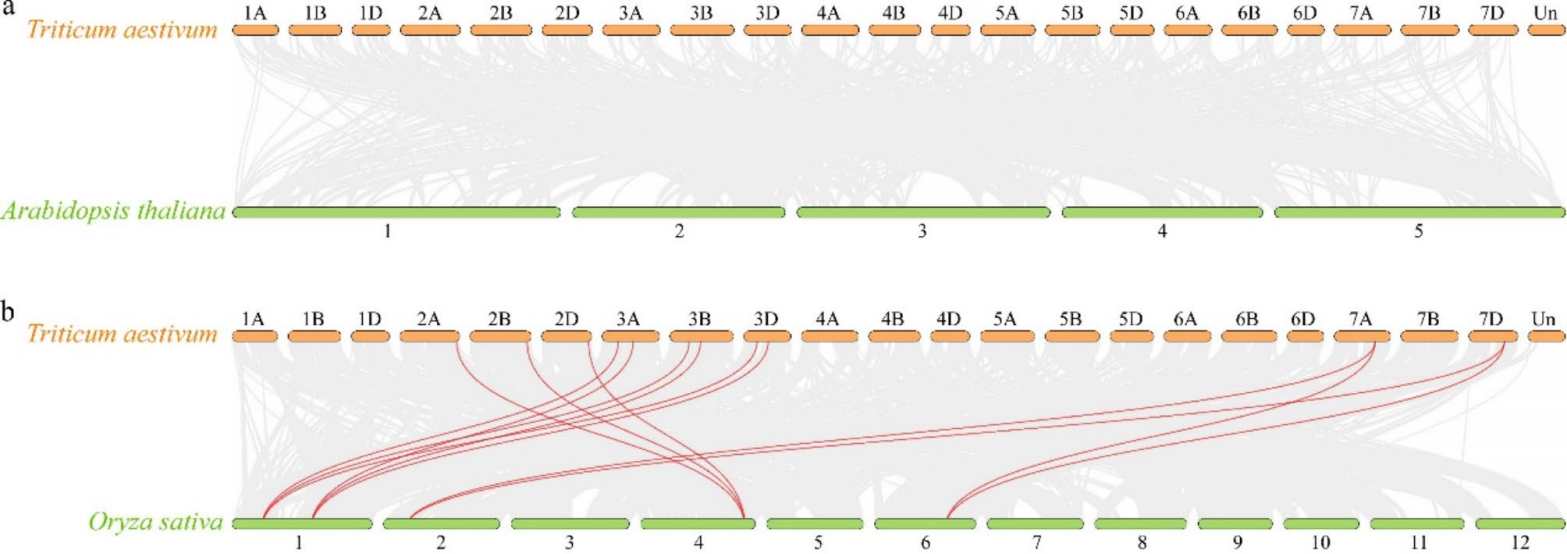
Syntenic relationships of the *UBA2* genes in wheat and two other species. (**a**) Syntenic relationships of *UBA2s* between wheat and *Arabidopsis thaliana*. (**b**) Syntenic relationships of *UBA2s* between wheat and *Oryza sativa*. Gray lines in the background indicate the synteny blocks of wheat and other species, while the red lines mark the syntenic *UBA2* gene pairs

To further study the evolutionary relationships of *TaUBA2* family, we performed syntenic analysis using TBtools to determine *TaUBA2* paralog gene pairs in *Triticum aestivum* and *TaUBA2* orthologous gene pairs between *Triticum aestivum* and *Arabidopsis thaliana*, as well as between *Triticum aestivum* and *Oryza sativa*. In our study, 17 paralogues were detected in *Triticum aestivum* (Table S2). There were no *TaUBA2* orthologous gene pairs were identified between *Triticum aestivum* and *Arabidopsis thaliana* (Fig. 6a), however, 13 orthologous gene pairs were detected between *Triticum aestivum* and *Oryza sativa* (Fig. 6b). These results showed that *UBA2* genes in *Triticum aestivum* were distantly related to those in *Arabidopsis thaliana*, whereas were closely associated with those in *Oryza sativa*. In genetics, the Ka/Ks ratio could clarify whether selective pressure acted on the plant genes [48]. On the whole, Ka/Ks greater than 1 indicates accelerated evolution with advantageous selection, Ka/Ks equal to 1 indicates neutral selection, and Ka/Ks less than 1 indicates purifying selection [47]. As shown in Table S2 and S3, the Ka/Ks ratios of *TaUBA2* paralogous gene pairs were less than one, and the Ka/Ks ratios of *TaUBA2* orthologous gene pairs were also less than one, these results indicated that purifying selection were more important during the *UBA2* family evolution. To further elucidate the evolutionary trends of *UBA2* family, we calculated the divergence time (T) based on the Ks values. The results illustrated that the divergence time of 17 paralogous gene pairs varied from 1.427 to 62.323 million years age (Mya), whereas the divergence time of orthologous gene pairs between *Triticum aestivum* and *Oryza sativa* ranged from 28.915 to 47.078 Mya (Table S2 and S3).

### Prediction and analysis of cis-acting elements in promoter regions of *TaUBA2s*

**Fig. 7:**
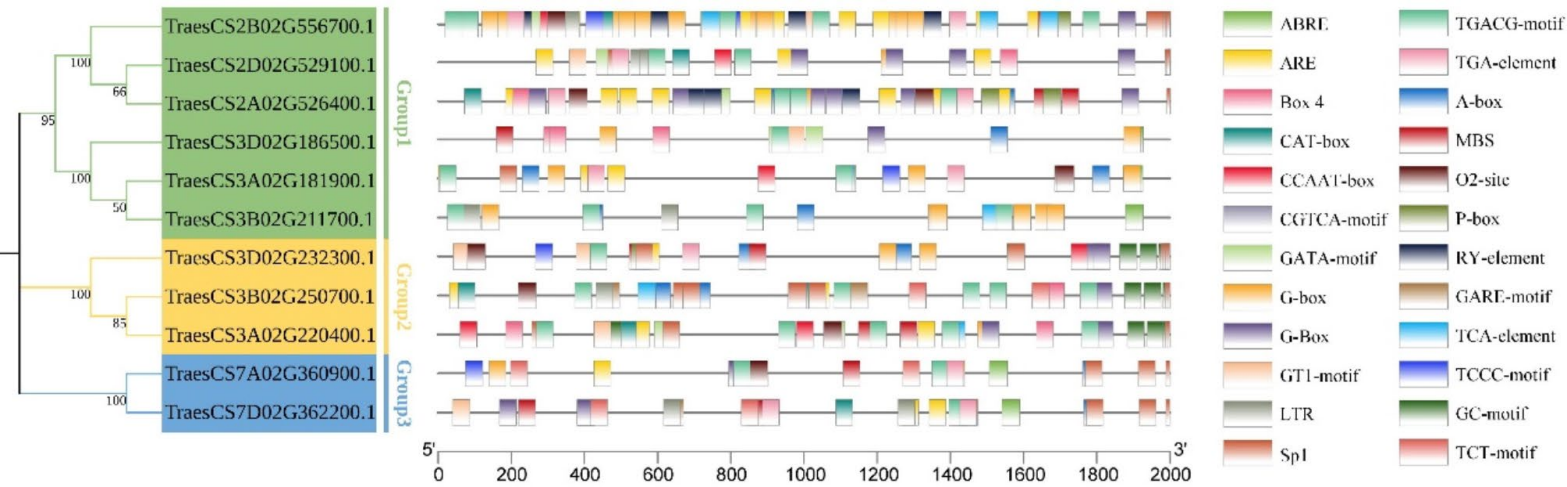
Prediction of cis-acting regulatory elements in *TaUBA2s*. Phylogenetic tree of TaUBA2s is shown on the left. The type and position of cis-acting elements predicted in *TaUBA2s* is shown in the middle. Different colored boxes represent different cis-acting elements. Names of cis-acting elements are diaplayed on the right

It is widely known that cis-acting regulatory elements in promoter regions are able to control the gene expression levels via binding to transcription factors [49, 50]. As previous reported, cis-acting regulatory elements participated in multiple biotic or abiotic stresses [51, 52]. To analysis the function of cis-acting regulatory elements of *TaUBA2* genes in biotic and abiotic stress, we detected the promoter regions of the eleven *TaUBA2* gene family members by the PlantCARE. A total of 1664 cis-acting elements were detected in *TaUBA2s*. These cis-acting regulatory elements in *TaUBA2s* were related to hormone response, environmental stress, promoter and enhancer elements, light response, development, and binding-site elements, which indicated that cis-acting regulatory elements of *TaUBA2s* were essential for wheat growth and development. The hormone response-related cis-acting regulatory elements, such as gibberellin (GA), auxin (IAA), salicylic acid (SA), methyl jasmonate (MeJA) and abscisic acid (ABA) were the most abundant, suggesting that hormone could significantly affected *TaUBA2* gene family. The TGACG- and CGTCA-motifs were involved in the response to MeJA, whereas the auxin-responsive element consisted of TGA-element. The SA-responsive element included the TCA-element. Additionally, the abscisic acid-responsive element (ABRE) was involved in the response to ABA. The environmental stress-related elements contained LTR and MBS, which participated in temperature and drought responses, respectively. In addition, we also detected cis-acting regulatory elements associated with light response in *TaUBA2s*, such as GT1-motif, G-box, TCT-motif, G-Box, GATA-motif, Box 4, TCCC-motif and Sp1. Moreover, the CAT-box element was involved in meristem expression, and the RY-element was related to seed-specific regulation (Fig. 7). Taken together, different *TaUBA2* gene family members included distinct numbers and types of cis-acting elements.

### RRM1 is essential for the subcellular distribution of TaUBA2C

**Fig. 8:**
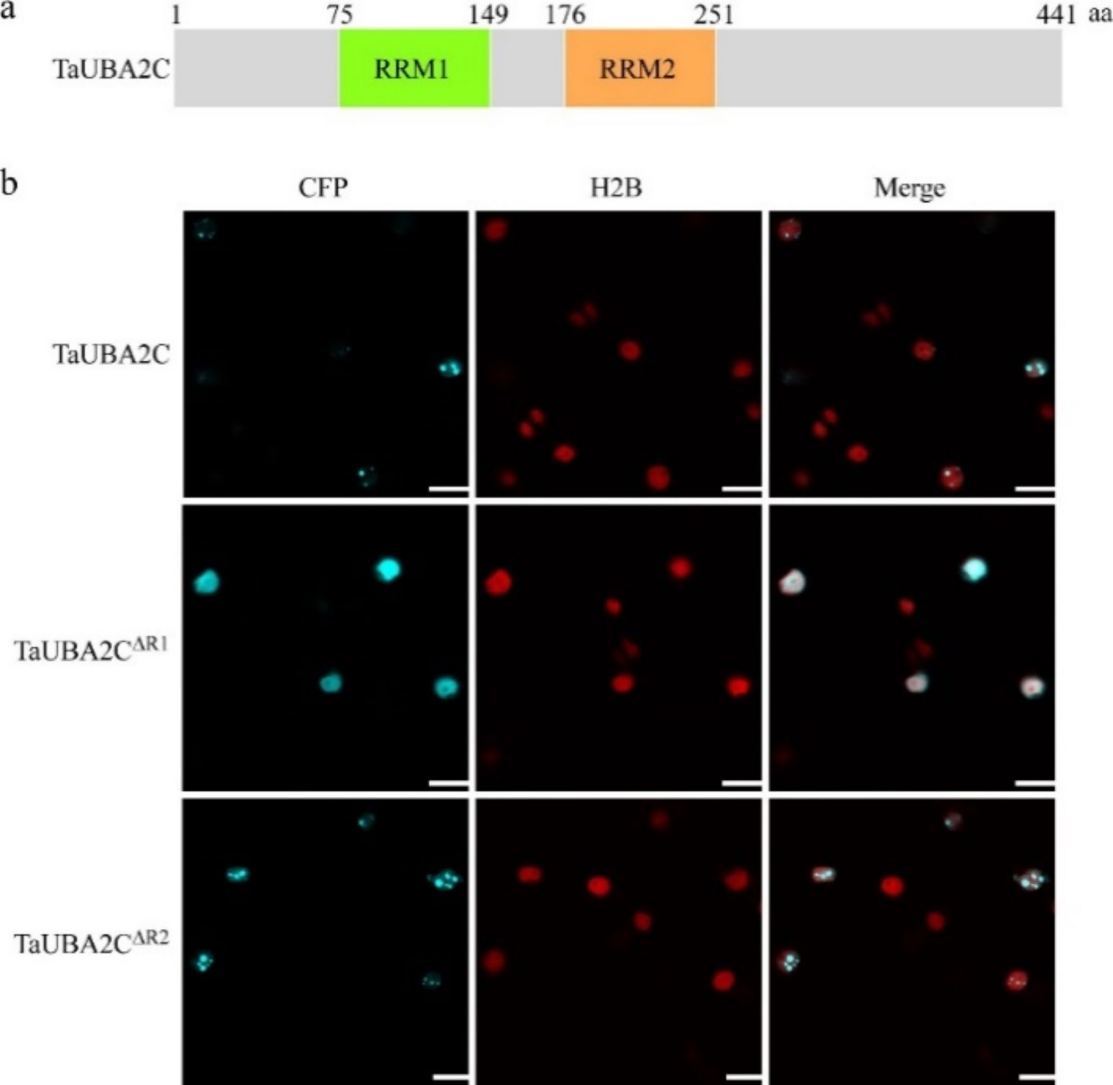
The first RNA-recognition motif of TaUBA2C is required for the nuclear speckle formation. (**a**) A schematic displays the distribution of domains in TaUBA2C. The numbers above the schematic represent the amino acid positions of different domains. RRM, RNA-recognition motif; aa, amino acids. (**b**) Subcellular localization of TaUBA2C-CFP and its mutants in H2B-RFP transgenic *N*. *benthamiana* leaves. Confocal imaging was performed at 60 h post infiltration (hpi). Scale bar = 20 μm

The function of proteins is closely related to their subcellular localization. It is reported that AtUBA2c (At3g15010) containing two RNA recognition motifs (RRMs) can form speckles in the nucleus, and the two RRMs are required for forming the nuclear speckles [28]. The TaUBA2 family member TraesCS3A02G220400.1 (named TaUBA2C) and AtUBA2c belong to the same subfamily (Group 2) in phylogenetic analysis (Fig. 2), meanwhile, TaUBA2C also contains two RRMs (RRM1:residues 75–149 and RRM2:residues 176–251) (Fig. 8a). In addition, previous study had confirmed that TaUBA2C could also form the nuclear speckles [29], however, the roles of these two RRMs of TaUBA2C in nuclear speckles formation was still unknown. To investigate which RRM is responsible for forming the nuclear speckles of TaUBA2C, we generated CFP-tagged TaUBA2C mutants with the RRM1 or RRM2 deletion (TaUBA2C^∆R1^-CFP and TaUBA2C^∆R2^-CFP). Then, we transiently expressed TaUBA2C-CFP, TaUBA2C^∆R1^-CFP, and TaUBA2C^∆R2^-CFP in the H2B-RFP transgenic *N*. *benthamiana* leaves via agroinfiltration methods, respectively. The CFP fluorescence in the leaf cells expressing TaUBA2C-CFP or its mutants were observed after 2 days inoculation using the confocal microscope. Consistent with previous report, TaUBA2C was located in the nucleus and presented in the form of speckles [29]. Meanwhile, we observed that TaUBA2C^∆R2^-CFP fusion proteins also presented as speckles in the nucleus, however, TaUBA2C^∆R1^-CFP fusion proteins were evenly distributed in the nucleus without speckles (Fig. 8b). These results indicated that the RRM1 of TaUBA2C was responsible for forming the nuclear speckles.

### The RRM1 and RRM2 of TaUBA2C are both required for inducing the cell death response

**Fig. 9:**
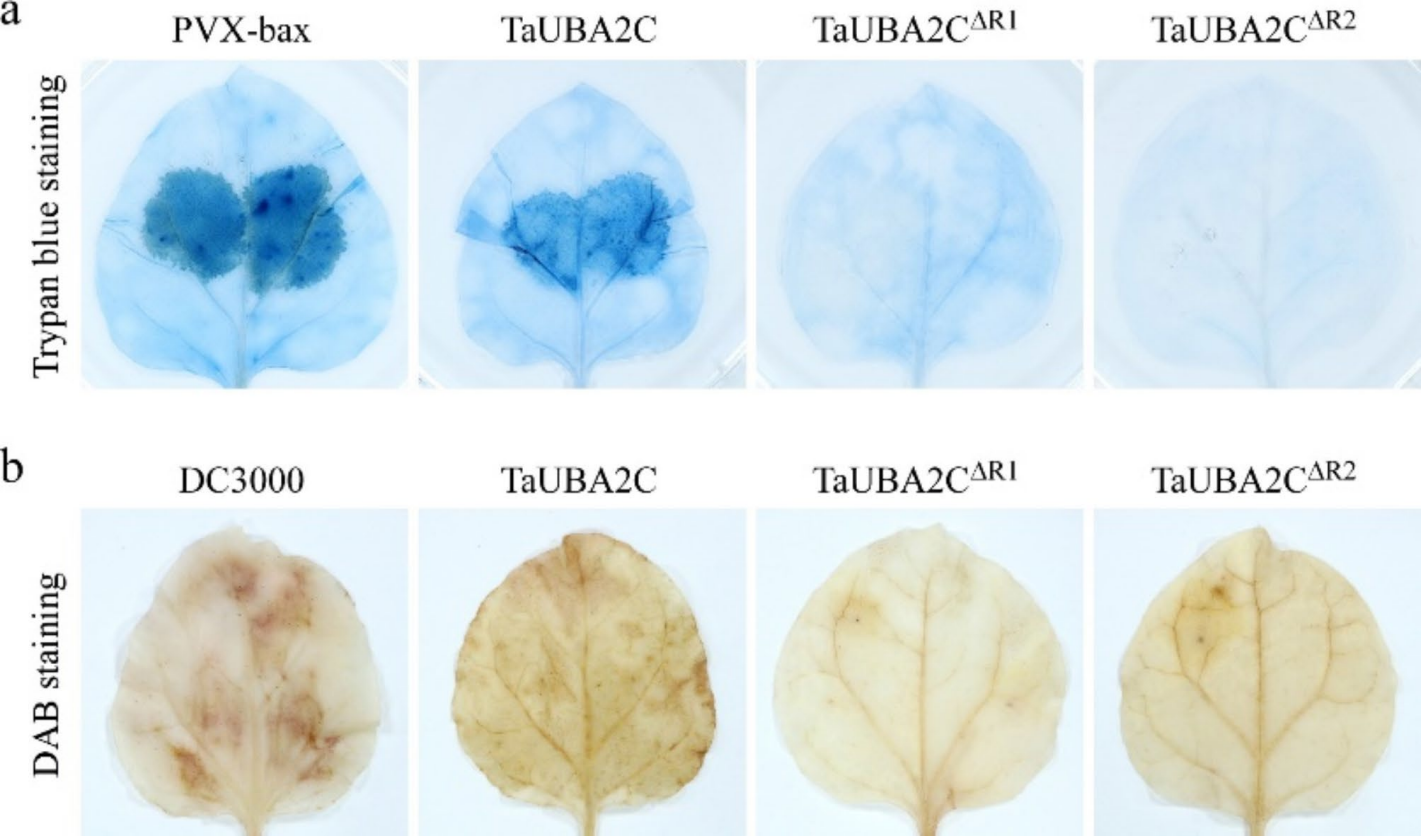
Both RRM domains are required for the cell death induction of TaUBA2C. (**a**) Cell death in *N*. *benthamiana* leaves expressing TaUBA2C or its mutants. The infiltrated leaves were stained with trypan blue solution at 5 days post agroinfiltration. The *N*. *benthamiana* leaf inoculated with PVX-bax served as a positive control. (**b**) H_2_O_2_ accumulation in assayed *N*. *benthamiana* leaves. By 5 dpi, the infiltrated leaves were stained with DAB solution. The *N*. *benthamiana* leaf infiltrated with the wild type Agrobacterium DC3000 served as a control

We previously reported that transient expression of TaUBA2C could induce the cell death response [29]. To further determine which RRM of TaUBA2C is responsible for inducing the cell death response, we transiently expressed TaUBA2C-Flag, TaUBA2C^∆R1^-Flag, and TaUBA2C^∆R2^-Flag in *N. benthamiana* leaves via agroinfiltration methods, respectively. The *N. benthamiana* leaves expressing PVX-bax were used as positive controls. After 5 days inoculation, we performed trypan blue staining analysis. Consistent with previous study, the areas expressing TaUBA2C-Flag fusion protein appeared cell death similar to PVX-bax expression. However, we observed that transient expression of the RRM1 or RRM2 deletion mutants, TaUBA2C^∆R1^-Flag and TaUBA2C^∆R2^-Flag, did not initiate cell death response (Fig. 9a). These results suggested that both RRM1 and RRM2 of TaUBA2C were necessary for the cell death response. Because TaUBA2C could induce H_2_O_2_ production, we then analyzed the roles of RRM1 and RRM2 of TaUBA2C on H_2_O_2_ production. The *N. benthamiana* leaves expressing TaUBA2C-Flag, TaUBA2C^∆R1^-Flag, and TaUBA2C^∆R2^-Flag were stained with DAB at 5dpi. The *N. benthamiana* leaf inoculated with DC3000 was used as positive control. The result showed that H_2_O_2_ had accumulated in the leaf expressing TaUBA2C-Flag as previous reported [29], however, we were unable to detect the H_2_O_2_ accumulation in *N. benthamiana* leaves expressing TaUBA2C^∆R1^-Flag and TaUBA2C^∆R2^-Flag, which indicating that both RRM1 and RRM2 of TaUBA2C were required for H_2_O_2_ production (Fig. 9b). Based on the above results, we concluded that both RRM1 and RRM2 of TaUBA2C were crucial for the biological function of TaUBA2C.

## Discussion

RNA binding proteins play crucial roles in RNA metabolism, such as synthesis, processing, transport, translation, stability and degradation of RNA, which in turn regulate gene expression, thereby participating in plant growth, development, environmental stress and plant immunity [21, 53, 54]. Although numerous previous studies have identified and characterized a variety of RBP members [2, 3, 11, 15, 21, 53–58], our understanding of wheat RBPs remains quite limited. Given the pivotal role of wheat in global food crop supply, we identified and characterized *TaUBA2* gene family, a subfamily within the RBP class, at the genomic level through bioinformatics tools [59]. This endeavor facilitates further investigation into the functions of *UBA2s* in plant growth and development. In our study, we systematically identified 11 TaUBA2 family members in wheat genome, and they were divided into three subfamilies, Group1, Group2 and Group3, according to their conserved domains (Fig. 1). We observed that the conserved domains, motif compositions, and exon-intron structures of the TaUBA2 family members were relatively conserved within each subfamily (Figs. 1 and 4b and c). Meanwhile, we predicted their protein spatial structures, and the results showed that there are significant differences in the protein structures of different subfamily, which support the proposed subfamilies, whereas the protein structures of the same subfamily are highly similar except Group3, suggesting that the two members (TraesCS7A02G360900.1 and TraesCS7D02G362200.1) of Group3 may have different functions (Fig. 3). Six *ZmUBA2* members in maize and four *OsUBA2* members in rice were identified via BLASTP, and the *Arabidopsis* UBA2 family only has three members, UBA2a, UBA2b, and UBA2c [2]. It is obvious that the wheat genome has the highest number of UBA2 members among the several species, with approximately three times as many members as *Arabidopsis* and *Oryza sativa*. This may be attributed to the fact that wheat is a complex allohexaploid (AABBDD) with a larger genome (about 17 Gb) [60]. Phylogenetic analysis showed that AtUBA2c (At3g15010) and TaUBA2C (TraesCS3A02G220400.1) belong to the same subfamily (Fig. 2), suggesting that they may have similar functions. Previous studies have shown that UBA2c in *Arabidopsis* is a flowering repressor and it can regulate flowering time through promoting FLM transcription [28]. Therefore, we speculate that TaUBA2C may be involved in wheat flowering.

Chromosomal locations and synteny analyses have been constructed to comprehensively investigate the relationships within the *TaUBA2* gene family. The result showed that 11 *TaUBA2* genes were unevenly distributed on the chromosome 2, 3, and 7 (Figure S1). Gene duplication events are essential for the expansion of gene families as well as the evolution or rearrangement of genomes, which is mainly attributed to tandem, segment, and transposition duplications, and they can help organisms increase functional diversity to adapt to various environments [48, 61–63]. In this study, ten collinear *TaUBA2* gene pairs were identified, indicating that segmental duplication events played important roles in the expansion of the *TaUBA2* gene family (Fig. 5). To gain insights into the evolutionary dynamics of *UBA2* genes, we conducted a comprehensive analysis of the Ka, Ks, and Ka/Ks ratios for both paralogous and orthologous gene pairs within the *UBA2* family. Our findings, presented in Tables S2 and S3, reveal that the Ka/Ks values for both sets of gene pairs consistently fell below 1. This observation underscores the prevalence of purifying selection in shaping the evolutionary trajectory of the *UBA2* gene family, indicating a significant role for this type of selective pressure in maintaining functional integrity across these genes. Previous studies have declared that orthologous analysis is an effective method for predicting unsuspected functions of homologous genes in different species, and orthologs usually have the same function [64, 65]. In this study, we identified 13 *UBA2* orthologous gene pairs between wheat and *Oryza sativa* (Fig. 6), suggesting that *TaUBA2s* and *OsUBA2s* may have similar function. Previous studies have shown that cis-acting regulatory elements participate in the regulation of gene transcriptional activity under phytohormones, photoreactions and various environmental stress [66]. Thus, we forecasted cis-acting regulatory elements of *TaUBA2s* to explore their possible biological functions. Our results showed that *TaUBA2s* promoter contained cis-acting elements related to hormone response, environmental stress, light response and so on, and different *TaUBA2* family members contained distinct numbers and types of cis-acting elements (Fig. 7). Therefore, we speculate that *TaUBA2s* may widely participate in various stress responses. Moreover, we found that *TaUBA2s* promoter also included cis-acting regulatory elements associated with development and metabolism, such as RY element, CAT-box, and O2-site. Overall, our results indicated that *TaUBA2s* may play critical roles in multiple aspects of wheat growth and development.

The accurate subcellular localization of proteins plays crucial roles in their activation and right function. For instance, the innate immune receptor RPM1 in plant cells is activated and functions on the plasma membrane [67]. The nuclear localization of *Arabidopsis* NPR1 is necessary for its regulation of PR gene expression [68]. AtRBP1-DR1 is localized in the cytoplasm to positively modulate the SA-mediated plant immunity [16]. CaRBP1, a pepper RNA-binding protein, can induce cell death response when it is located in the cytoplasm, moreover, N-terminal region of CaRBP1 is required for the cytoplasmic localization [69]. AtUBA2c, an RBP which contains two RRMs, is localized in the nuclear speckles, and the two RRMs are required for forming the nuclear speckles [28]. Our previous study has declared that TaUBA2C was also localized in the nuclear speckles [29], however, the roles of these two RRMs of TaUBA2C in nuclear speckle formation was still unknown. To investigate which RRM is responsible for forming the nuclear speckles of TaUBA2C, we performed RRM domain deletion analysis. Unlike AtUBA2c, RRM1 is the key domain for the nuclear speckle formation of TaUBA2C, whereas the absence of RRM2 does not affect the subcellular localization of TaUBA2C (Fig. 8). Next, we explored which RRM is necessary for the biological function of TaUBA2C. Trypan blue staining and DAB staining results suggested that both RRM1 and RRM2 are required for inducing cell death response and H_2_O_2_ production (Fig. 9). This is consistent with the results observed in *Arabidopsis* UBA2c, where the two RRMs of AtUBA2c are necessary for its biological function [28]. In the future, it will be very interesting to study why the RRM2 domain of TaUBA2C does not affect its subcellular localization but is crucial for its function.

Taken together, through this study, we identified and characterized the wheat UBA2 family via the genome-wide analysis. Meanwhile, we have confirmed that the two RRM domains of TaUBA2C are essential for its biological function. We also found that the UBA2 family is likely to play an important role in the development and metabolism of wheat, and may be involved in wheat flowering. Our results provide some reference for the subsequent functional studies of UBA2 family members in wheat. Further research is needed to elucidate the biological functions of the UBA2 family in wheat growth and development.

## Conclusions

In this study, we identified 11 members of the *UBA2* family in wheat, which could be categorised into three clades (Group1, Group2, and Group3). *TaUBA2s* within the same subfamily had relative conserved protein domains, motifs, and gene structures. Additionally, *TaUBA2* gene family members unevenly distributed on the wheat chromosomes with 10 collinear *TaUBA2* gene pairs, suggesting that segmental duplications played important roles in the expansion of *TaUBA2* family. Cis-acting elements analysis showed that *TaUBA2s* participated in hormone response, development, light response, metabolism, and response to environmental stress. RRM domain deletion analysis implied that RRM1 is necessary for the nuclear speckle formation of TaUBA2C, and the two RRMs are required for inducing cell death response and H_2_O_2_ production. Our results contribute to a comprehensive understanding of the *TaUBA2* family and provide reference for subsequent functional studies of *TaUBA2* family members.

## Methods

### Genome-wide identification of *TaUBA2* family

The amino acid sequences of *Arabidopsis* UBA2 family members (At3g56860, At2g41060, and At3g15010) obtained from the *Arabidopsis* Information Resource (https://www.arabidopsis.org; accessed on 11 March 2024) were used as queries to identify UBA2 members in wheat, rice, and maize through BLASTP using the Ensemble Plants database (http://plants.ensembl.org/; accessed on 11 March 2024) (E-value < 10^− 5^). After removing the redundant sequences, candidate proteins were further screened with the Pfam database (http://pfam.xfam.org/; accessed on 11 March 2024) [70] and NCBI Batch Web CD-Search Tool (https://www.ncbi.nlm.nih.gov/Structure/bwrpsb/bwrpsb.cgi; accessed on 11 March 2024) [71]. Detailed information of TaUBA2s, such as chromosomal location, CDS length, protein size and the number of exons was obtained from the Ensembl Plants. The MW and pI of TaUBA2 proteins were analyzed using ExPASy (https://web.expasy.org/compute; accessed on 12 March 2024) [72].

### Multiple sequence alignment and phylogenetic analysis

Multiple sequence alignment of AtUBA2s, TaUBA2s, OsUBA2s, and ZmUBA2s was carried out using ClustalW in MEGA7.0 with default parameters [73, 74]. Then, the phylogenetic tree of UBA2 family was generated based on the neighbor-joining (NJ) method by 1000 bootstrap tests. The data processing adopted pairwise deletion, and the Poisson distribution was used for tree-building model.

### Structural prediction of TaUBA2 proteins

The spatial structure of the TaUBA2 proteins was predicted through the automated SWISS-MODEL homology modeling server (https://swissmodel.expasy.org/; accessed on 15 March 2024) [43].

### Conserved-domain, motif, and gene structure analysis of *TaUBA2s*

The protein sequences of TaUBA2 were imported into the NCBI Batch CD-Search tool (https://www.ncbi.nlm.nih.gov/Structure/bwrpsb/bwrpsb.cgi; accessed on 16 March 2024), and the conserved domain data were generated and visualised via TBtools. Gene annotation files of wheat were obtained from the Ensembl Plants Database (http://plants.ensembl.org/; accessed on 16 March 2024) [75], then, we analyzed the gene structure of *TaUBA2* family through TBtools Gene Structure View [76]. The motifs of TaUBA2 were analyzed using the MEME online tool (https://meme-suite.org/meme/tools/meme; accessed on 16 March 2024) with a maximum selection of 12 motifs [77], and the results were visualized via TBtools software [76].

### Chromosomal locations and synteny analysis

To study the distribution of *TaUBA2s* in wheat chromosomes and gene duplication events, the related data of the wheat genome was obtained from the Ensembl Plants database (http://plants.ensembl.org/; accessed on 18 March 2024). Then, we analyzed the chromosomal location and synteny relationship of *TaUBA2s* using TBtools [76]. The Ka/Ks values were calculated through TBtools, and the divergence times (T) were calculated according to T = Ks/(2 *×* 9.1 *×* 10^*−* 9^)Mya [47].

### Prediction of cis-acting elements of *TaUBA2s*

The 2000 bp upstream sequences of each *TaUBA2* gene were obtained from the Ensemble Plants database, and the acquired sequences were used to analyze cis-acting elements via PlantCARE software (http://bioinformatics.psb.ugent.be/webtools/plantcare/html/; accessed on 19 March 2024) [78].

### Trypan blue staining

The agroinfiltrated *Nicotiana benthamiana* leaves were collected, and analyzed for cell death using trypan blue staining. As previously described [29], the collected leaves were soaked in trypan blue solution and boiled for 3–5 min. The stained leaves were de-stained via 2 ~ 3 rinses in chloral hydrate (2.5 g/ml) solution followed by photographing.

### DAB staining

The accumulation of H_2_O_2_ in the agroinfiltrated leaves was analyzed using 3,3’-diaminobenzidine (DAB) staining method as previously reported [79]. Briefly, the collected leaves were soaked in DAB (1 mg/mL) staining solution (Sigma) overnight followed by 3 ~ 5 de-staining in absolute ethanol.

## Electronic supplementary material

Below is the link to the electronic supplementary material.

Supplementary Material 1

Supplementary Material 2

Supplementary Material 3

Supplementary Material 4

Supplementary Material 5

## Data Availability

The data included in this study and the additional files are available. The sequences of Arabidopsis thaliana, Triticum aestivum, Oryza sativa and Zea mays are available in the Ensemble Plants database (http://plants.ensembl.org/index.html).
