# Supplementary material for: Genome-wide identification and analysis of the UBA2 gene family in wheat (Triticum aestivum L.)
Source: BMC Genomics. 2025 Feb 22;26:180. doi: 10.1186/s12864-025-11352-z (PMC11847341; doi:10.1186/s12864-025-11352-z)

Additional file 1: Figure S1. Chromosome locations of *TaUBA2* genes. Cylinders represent the wheat chromosomes. The green font represents Group1, the orange font represents Group2, and the blue font represents Group3.


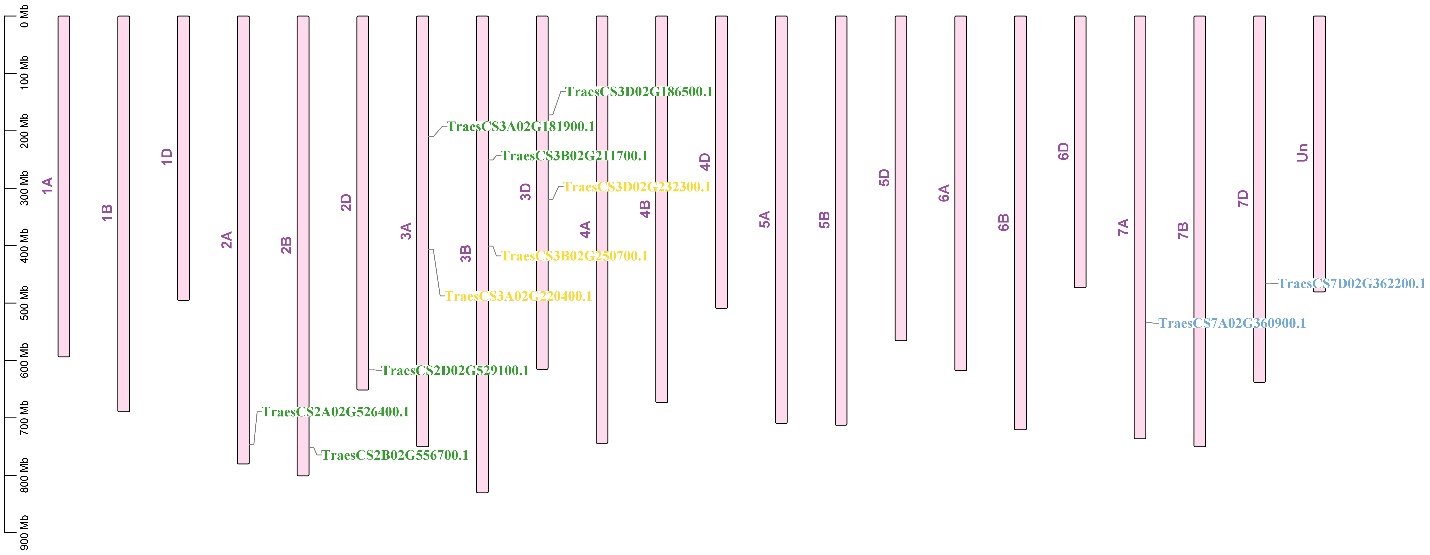

Supplement: Supplementary file 1 — Supplementary Material 1 [file 12864_2025_11352_MOESM1_ESM.docx]
